# The Transformation of 0-D Carbon Dots into 1-, 2- and 3-D Carbon Allotropes: A Minireview

**DOI:** 10.3390/nano12152515

**Published:** 2022-07-22

**Authors:** Lerato L. Mokoloko, Roy P. Forbes, Neil J. Coville

**Affiliations:** DSI-NRF Centre of Excellence in Catalysis and the Molecular Sciences Institute, School of Chemistry, University of the Witwatersrand, Johannesburg 2050, South Africa; 562061@students.wits.ac.za (L.L.M.); roy.forbes@wits.ac.za (R.P.F.)

**Keywords:** carbon allotropes, carbon dots, carbon nanotubes, graphene-based nanosheets, porous carbon frameworks

## Abstract

Carbon dots (CDs) represent a relatively new type of carbon allotrope with a 0-D structure and with nanoparticle sizes < 10 nm. A large number of research articles have been published on the synthesis, characteristics, mechanisms and applications of this carbon allotrope. Many of these articles have also shown that CDs can be synthesized from “bottom-up” and “top-down” methods. The “top-down” methods are dominated by the breaking down of large carbon structures such as fullerene, graphene, carbon black and carbon nanotubes into the CDs. What is less known is that CDs also have the potential to be used as carbon substrates for the synthesis of larger carbon structures such as 1-D carbon nanotubes, 2-D or 3-D graphene-based nanosheets and 3-D porous carbon frameworks. Herein, we present a review of the synthesis strategies used to convert the 0-D carbons into these higher-dimensional carbons. The methods involve the use of catalysts or thermal procedures to generate the larger structures. The surface functional groups on the CDs, typically containing nitrogen and oxygen, appear to be important in the process of creating the larger carbon structures that typically are formed via the generation of covalent bonds. The CD building blocks can also ‘aggregate’ to form so called supra-CDs. The mechanism for the formation of the structures made from CDs, the physical properties of the CDs and their applications (for example in energy devices and as reagents for use in medicinal fields) will also be discussed. We hope that this review will serve to provide valuable insights into this area of CD research and a novel viewpoint on the exploration of CDs.

## 1. Introduction

Carbon is one of the most widely exploited elements that have been used in nanotechnology [1,2]. This is no surprise, as carbon has many fascinating characteristics, including its most important ability to bond with itself, which has led to carbon being able to form 0-, 1-, 2- or 3-D naturally occurring and synthetically made allotropes. These carbon allotropes include graphite, diamond, graphene, fullerene, carbon nanotubes (CNTs), carbon nanofibers (CNFs), carbon black (CB), carbon nano-onions (CNOs), carbon spheres (CSs) and hollow carbon spheres (HCSs). Due to their remarkable chemical, mechanical, electrical and thermal properties, these allotropes have been used in drug delivery, electronics, composite materials, sensors, energy storage and conversion and field emission devices [1,2,3,4]. In addition, the various carbon allotropes can be derived from a range of highly abundant starting materials, including renewable sources and waste materials, using a wide variety of synthesis strategies. This makes the use of carbon even more important, especially towards green and sustainable nanoscience and nanotechnology applications [5,6,7].

**Figure 1:**
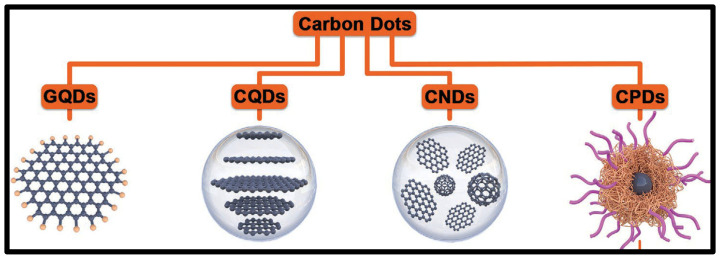
The proposed representative structures of a graphene quantum dot (GQD), carbon quantum dot (CQD), carbon nanodot (CND) and carbonized polymer dot (CPD). Adopted with permission from Wiley-VCH Verlag [12].

Another relatively new carbon allotrope that has been made and studied is the carbon dot (CD). CDs are a group of carbon nanomaterials that are made of zero-dimensional (0-D) nanoparticles (<10 nm), and because of their small size they show intrinsic fluorescence properties [8,9,10,11]. These carbons include graphene quantum dots (GQDs), carbon quantum dots (CQDs), carbon nanodots (CNDs) and carbonized polymer dots (CPDs) [11,12,13,14]. The different CDs are shown in Figure 1. All of these CDs show excellent optical properties, water dispersibility, biocompatibility, resistance to photobleaching and low toxicity, and hence these materials have been used in bioimaging, catalysis, sensing and drug delivery [6,15]. The increased attention given to CDs stems from their potential to replace traditional metal-based quantum dots (QDs), especially in the aforementioned applications [16,17,18]. This is because their competitive fluorescence properties are not associated with toxicity, environmental concerns and biohazard issues [19,20].

The CDs were first reported in 2004 after they were serendipitously discovered during the purification of single-walled carbon nanotubes using gel electrophoresis [21,22]. Then, in 2006, CDs were synthesized via the laser ablation of a targeted graphite powder and cement mixture surface [23]. Since then, CDs have been made via hydrothermal and solvothermal synthesis [24], electrochemical methods [25,26] and microwave-assisted synthesis [27]. CDs can also be made using “top-down” methods using larger-sized carbon allotropes such as carbon nanotubes (CNTs), graphite, carbon nanofibers (CNFs), carbon black and candle soot [28,29]. This is achieved by breaking down these larger carbon allotropes using strong oxidizing agents such as nitric acid or sulfuric acid [30]. They can also be made from carbon-containing molecules using a “bottom-up” method. These carbon sources range from carbohydrates such as sugar, sucrose and starch [31] to polymers (e.g., amino acids), biomass and municipal waste materials, such as fruits peels, hair and urine [32,33].

Details on the synthesis methods [15,19,32,34,35,36,37,38], yield [34,35,36], size [11,39], physicochemical properties [29,40,41,42,43] and applications [9,14,30,32,44,45,46,47,48,49,50,51,52] of the CDs have been extensively reported. Therefore, details on these topics may be found in other reports and will only be briefly mentioned in this review.

Of the two synthesis strategies, the “bottom-up” strategies are preferred because they show far better potential for the facile synthesis of CDs at low cost and large scale [5,53]. However, it is important to note that the CDs produced via the “top-down” approach allow for better structural control and the use of easier purification methods compared to the “bottom-up” approaches [53]. Additionally, the CDs obtained from a “top-down” method obtain some of the physical and chemical properties from the carbon allotropes from which they are derived. For example, graphene quantum dots (GQDs) derived from breaking down graphene oxide (GO) can inherit the crystallinity of the graphene oxide [54].

The conversion of one carbon allotrope to another via chemical or mechanical methods is not specific to the preparation of CDs from dimensional carbon allotropes. It is well-known that graphene nanolayers can be derived from graphite [55,56,57,58], and that graphite can be made from graphene [59]. Graphene can also be synthesized from fullerene [60,61,62]. Furthermore, fullerene can be exfoliated to form an atomic-scale 2-D polymeric network [63]. Reports have also indicated that graphene and graphene nanoribbons can be prepared by unravelling CNTs [64,65,66,67,68,69,70]. Further, CNTs can be grown on a CB substrate [71,72]. Other examples include the transformation of CB to graphene-oxide-like nanosheets [73,74] and HCSs [74]. Graphite can also be converted to diamonds, and vice versa [75,76,77]. Thus, the conversion of one allotrope to another is a well-known procedure for making high-quality carbon allotropes with size control [69,72,78,79].

The ability to transform large carbon allotropes to smaller carbon entities suggests that the reverse should also be possible. Indeed, some recent studies have also shown that CDs can be used as carbon substrates in a “bottom-up” process to make CNTs, graphene-based nanosheets (GNSs) and porous carbon frameworks (PCFs). This route from CDs creates an interesting synthesis route to make novel 1-, 2- and 3-D carbon allotropes. Indeed, Chen et al. have summarized the early work on the synthesis of CDs and composites and reported on their unusual properties [80]. This conversion route from CDs could potentially be a simpler and more cost effective option than the alternate routes for the controlled synthesis of many carbon allotropes, since there is a huge library of CDs that have been made, many from renewable resources [6,33]. Further, while some applications of these CD-derived carbon allotropes have been reported, very little detailed work has been done to exploit these materials [20,81,82]. In this review, we describe the methods that have been used to convert CDs to other allotropes and also provide details on the proposed mechanisms for the evolution of 0-D CDs to larger carbon allotropes (1-, 2- and 3-D carbon allotropes). A brief discussion of the applications of the final allotropes made from the CDs is given.

## 2. Synthesis and Structure Relationship of Various CDs

Most CDs are typically made up of ordered or disordered graphitic flakes that constitute a carbon core, with the end carbon atoms terminated by hydrogen atoms or functional groups containing oxygen or nitrogen [6,83]. The final structure and characteristics of the CDs are dependent on the type of synthesis approach employed in their fabrication. As previously mentioned, the CDs can be synthesized using either the “top-down” or the “bottom-up” synthesis approaches. The “top-down” synthesis approaches typically produce GQDs, which consist of a graphitic core encompassed by carbons containing oxygen-based functional groups. In contrast, “bottom-up” approaches, which involve the building up of small organic molecules and polymers via carbonization, are commonly used to make CQDs, CNDs and CPDs [14] (Figure 1). This approach results in the synthesis of an amorphous core of pure sp^3^-type carbon or a core made with carbons with a range of sp^3^/sp^2^ carbon ratios, with the core terminated by carbon atoms with various functional groups. The CPDs are typically synthesized using polymers containing hydroxyl, carboxyl and amino groups, and possess an amorphous core terminated by polymer chains [84]. CQDs and CNDs on the other hand are typically built from carbohydrates and organic acids (e.g., ascorbic and citric acid). The CQDs and CNDs can be easily transformed into CPDs via the attachment of polymer moieties to their surfaces [5]. Biomass-derived precursors are also used in the “bottom-up” and “top-down” syntheses of different CD products [33].

**Figure 2:**
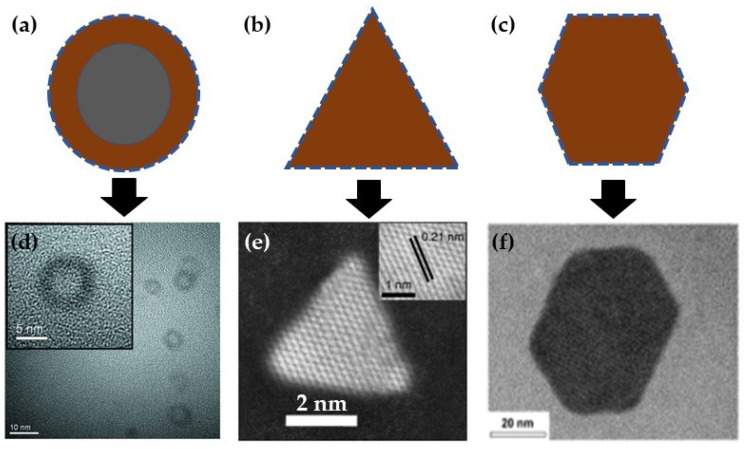
Illustrations of the shapes and TEM images of (**a**,**d**) hollow carbon dots (HCDs, adopted with permission from [85], Elsevier BV), (**b**,**e**) triangular carbon dots (triangular CDs, adopted with permission from [87], Springer Nature) and (**c**,**f**) single-crystal hexagonal graphene quantum dots (adopted with permission from [88], American Chemical Society).

Other forms of CDs include the hollow carbon dots that contain a functionalized amorphous shell formed from either a “top-down” or “bottom-up” approach [85,86]. Highly graphitized and triangular CDs can also be obtained using a “bottom-up” synthesis approach [87]. Single-crystal hexagonal graphitic CDs with a controlled morphology can also be obtained via a “bottom-up” synthesis [88]. Figure 2 below shows these differently structured CDs. More recently, CDs have been made from carbon nitrides such as cyanoethynyl quantum dots and beta carbon nitride carbon dots [89,90]. Based on these discoveries, it is, therefore, safe to predict that many other derivatives of CDs will be discovered in the future.

The unique structures of CDs capped with surface functional groups (oxygen- or nitrogen-based groups) allows for the easy modification of their physical, chemical, optical and electronic properties [8,91]. These surface functional groups have also been shown to be important for the knitting together of CDs to form other carbon allotropes (see below).

## 3. Synthesis of Larger Carbon Allotropes

**Figure 3:**
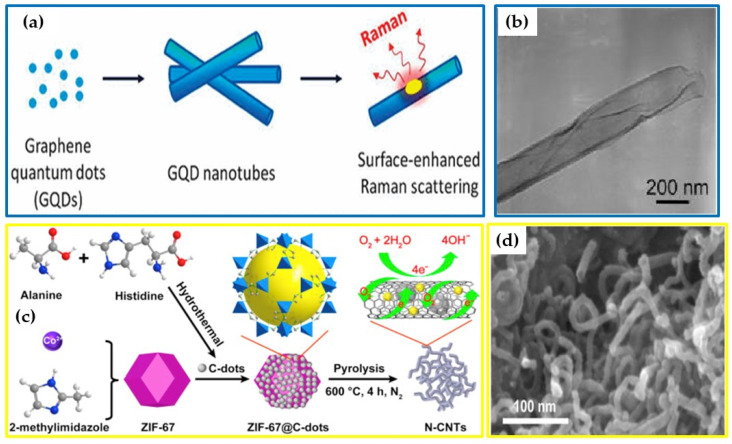
Illustrations of the formation of 1-D carbons from 0-D CNTs: (**a**,**b**) CNTs (adapted with permission from [100], copyright 2012, American Chemical Society) and N-CNTs; (**c**,**d**) N-CNTs (reproduced with permission from [101], copyright 2021, Elsevier BV).

Several different methods have been used to synthesize CNTs, graphene-based nanosheets (GNSs) and porous carbon frameworks (PCFs) from CD precursors. These routes, as well as the physicochemical properties of the resulting products with respect to their CDs precursors, are described below. Data on these processes are summarized in Table 1, and some examples of these routes are shown in Figure 3.

### 3.1. CDs Used as Precursors to Make 1-D Carbon Allotropes

CNTs have been studied in detail since their synthesis and initial study by Iijima in 1991 [92]. CNTs are described as 1-D carbon allotropes made with predominantly sp^2^-type carbon, rolled into a tubular structure, with typical diameters and lengths in the nano and micro or macro ranges, respectively [93,94]. CNTs are known for their excellent mechanical, electrical and optical properties, and have been widely explored for use in energy storage, catalysis, sensors and air filtration [95,96]. The traditional synthesis methods used to make CNTs involve arc discharge, laser evaporation, catalytic pyrolysis and the flame method, and these methods have been described in detail elsewhere [96,97]. While it is well known that CNTs can be cut down into CDs via chemical oxidation [98,99], the ability of CNTs to be built up from CDs has been less studied.

Cheng et al. showed that CDs can be transformed into CNTs via the electrophoresis deposition of CDs within a nanoporous anodic aluminum oxide template. The CDs were first synthesized via “top-down” electrochemical oxidation of graphene. The CDs were classified as graphene quantum dots (GQDs) and contained particles with an average size range of 3–5 nm [100]. The nanotubes obtained via the electrophoresis-aided transformation of the CDs were named graphene quantum dot nanotubes (GQD-NTs). A scheme summarizing the synthesis and TEM images of these GQD-NTs is shown in Figure 3a,b. These GQD-NTs have similar features to those associated with traditional CNTs, such as having a hollow and tubular morphology. The average diameter range of these GQD-NTs was found to approximately 200–300 nm. The size and morphology of these GQD-NTs was highly dependent on the morphology of the AAO template. Interestingly, the formed GQD-NTs’ structure still showed the individual CDs to be interconnected with an estimated gap of about 1 nm between the particles. The XPS studies revealed the presence of hydroxyl and carboxyl functional groups on the GQDs and GQD-NTs’ surfaces. They also revealed a decrease in the O/C ratio from 26.4 to 11.6 for the GQDs and GQD-NTs, respectively. This suggested that the GQD nanoparticles “merged” during the removal of the oxygen functional groups to form GQD-NTs on the AAO template.

Another study by Niu et al. showed that nitrogen-doped CNTs (N-CNTs) could be grown from a mixture of N-CDs and a metal–organic framework, ZIF-67 [101]. In this study, N-CDs were synthesized from a “bottom-up” hydrothermal reaction using alanine and histidine as precursors to give highly crystalline CDs with average particles sizes of approximately 10 nm. The ZIF-67 and N-CDs were mixed and reacted at 600 °C. The influence of the ZIF-67–N-CD ratios (30:1, 20:1 and 10:1) and reaction times (from 1 to 4 h) on the morphology of the product were investigated. The optimum reaction conditions for N-CNT formation were found to be a 20:1 (ZIF-67–N-CDs) ratio, pyrolyzed at 600 °C for 4 h (see reaction scheme in Figure 3c). Under these conditions, the N-CNTs that formed (Figure 3d) had an average diameter and length of about 15 nm and 300 nm, respectively. The N-CDs were responsible for reducing the cobalt (Co^2+^/^3+^) ion in the ZIF-67 into metallic Co (Co^0^). The N-CNTs were then grown on the Co catalyst surface during the carbonization of the organic residue of the ZIF-67 and the N-CDs under high temperature.

### 3.2. CDs Used as Precursors to Make 2-D Carbon Allotropes

Graphene-based nanosheets (GNSs) are an interesting group of 2-D and 3-D carbon allotropes consisting of a single or a few layers of sp^2^-type C atoms. These allotropes are known for their excellent mechanical properties, high surface area and versatile surface chemistry [102,103,104]. These attributes make them suitable for mechanical, optical, electrical and environmental applications, such as water purification [104]. GNSs can be made from pristine graphene, graphene oxide (GO) and reduced graphene oxide (rGO) starting materials. Pristine graphene consists of polycyclic aromatic rings forming a single layered nanosheet. In GO, the nanosheets are bonded to oxygen-based moieties, creating defects within the nanosheets. The C/O ratio is higher in rGO than in GO, and the structure has less defects [105]. GNSs are usually synthesized from “top-down” mechanical, chemical, thermal and electronic routes and via the solvent- or surfactant-based exfoliation of graphite. The “bottom-up” growth from molecular substrates has also been achieved, using the CVD method and via the graphitization of silicon carbide [106,107,108,109,110,111,112].

CDs with different morphologies and properties have been synthesized from graphene [100] and GO [39,79]. Other studies have shown that both CDs and GO/rGO can be obtained simultaneously using the same synthesis method. For example, Dong et al. showed that GQDs and GO can be made via the “bottom-up” carbonization of citric acid at 200 °C for 30 min and 2 h, respectively [113]. Another study by Muthurasu et al. showed that GQDs and rGO can be obtained via the electrolysis of a graphite rod at a constant current density of 100 mA cm^−2^ for 3 h [114]. The resulting solution was divided into a supernatant and precipitate, where the supernatant contained GQDs and the precipitate contained the rGO. Different CDs have also been used as carbon substrates to make a variety of 2-D sheets with similar properties to those of GNSs.

Chen et al. showed that CQDs can self-assemble into layered carbon sheets when freeze-dried [115]. The CQDs with average particle sizes of approximately 7 nm were synthesized by decomposing fullerene (C60) in potassium hydroxide. The CQDs were then dispersed in water and the solution was freeze-dried to give layers of interconnected CQDs at the ice–crystal–water interface. The CQDs were interconnected via oxygen functional group interactions on the CQDs’ surfaces. However, these layers rapidly dissociated into CQDs upon redispersion in water. To obtain the permanent layered carbon sheets, the CQDs were annealed at 600 and 800 °C for 30 min. At 800 °C the layered carbon sheets resembled a graphene morphology. However, there was no evidence of the hexagonal lattice fringes typical of graphene. The results from the XPS analysis showed an increase in the C/O ratio from 2.15 for CQDs to 3.14 for the annealed sample, with a significant decrease in the presence of the carbonyl functional groups, as confirmed by the FT-IR data. The study also showed that the layered carbon sheets contained a porous surface.

Hou et al. was able to obtain 2-D layered carbon nanosheets through the thermal treatment of CDs in the presence of metal ions [116]. In this study, CDs were obtained by mixing an acetaldehyde solution and NaOH at room temperature and pressure, and then allowing the mixture to stand for 3 days. The CDs had average particle sizes ranging between 3 and 5 nm. To obtain the 2-D carbon sheets, the CDs were combined with NaH_2_PO_4_, and then the mixture was thermally treated at temperatures between 400 and 900 °C. The transformation of the CDs to a 2-D carbon was observed to depend on both the temperature and the amount of NaH_2_PO_4_ used in generating the structure of the resulting sheets. The NaH_2_PO_4_ acted as a P-dopant and a source of Na^+^, and the Na^+^ metal ions catalyzed the transformation of CDs to nanosheets. The Na^+^ also controlled the sheet thickness. At 400 °C, the CDs transformed into carbon chunks, then finally at 900 °C they transformed into 2-D ultrathin sheets with lattice fringes of 0.42 nm. It was also observed that an increase in temperature increased the amount of P incorporated into the 2-D structures. On the other hand, without the NaH_2_PO_4_, the CDs only aggregated to form carbon chunks, and even at 900 °C no 2-D ultrafine nanosheets were observed. There was no significant difference in the C/O ratios of the samples, but the FT-IR spectra showed a significant decrease in the C=O bond intensity with increasing temperature. The authors proposed that the Na^+^ ions catalyzed the decomposition of the C-O bonds, thereby increasing the amount of C species in the system. Under heat treatment, these C species then combined to form C-C bonds that assembled into the layered carbon nanosheets.

**Figure 4:**
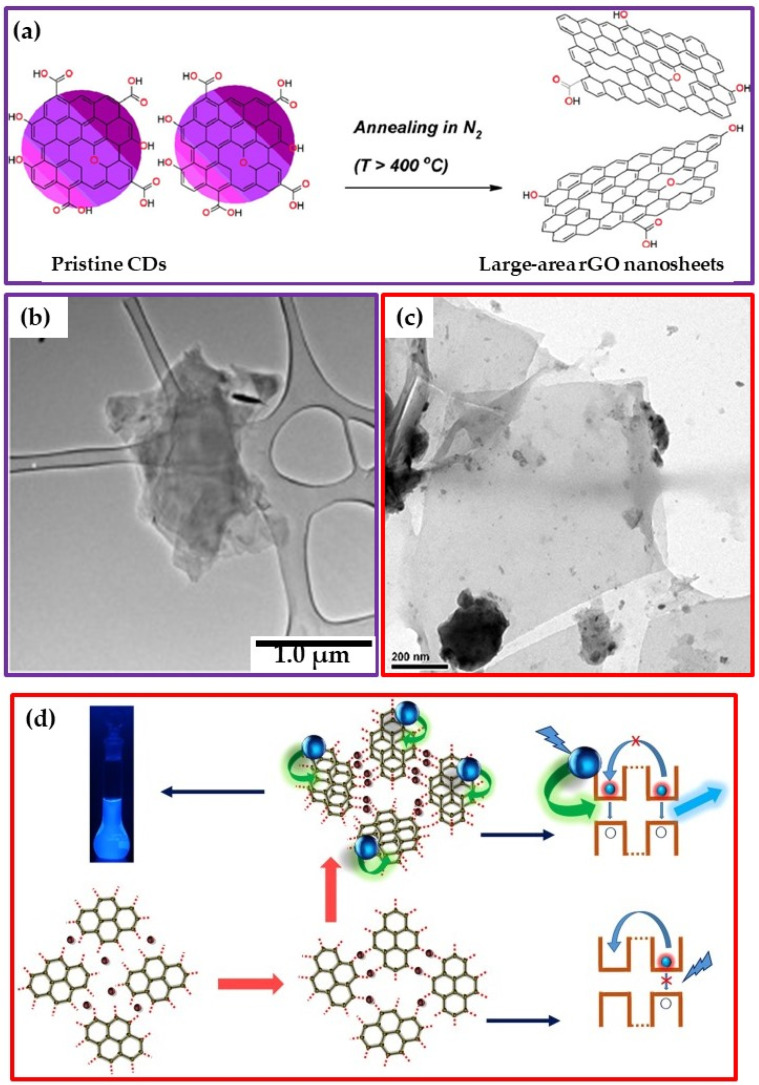
Schematic representation of the synthesis procedure and TEM images of the resulting product formed during the conversion of 0-D CDs to 2-D graphene-like nanosheets using a (**a**,**b**) high-temperature (> 300 °C) treatment without a catalyst (reproduced with permission from [117], copyright 2021, Elsevier BV) and (**c**,**d**) lower-temperature (180 °C) treatment in the presence of a metal catalyst (reproduced with permission from [118], copyright 2017, Springer Nature).

More recently, it was observed that the CDs formed 2-D graphene-like sheets without the need for a metal ion catalyst. In the study, the CDs were obtained via the microwave-assisted “bottom up” carbonization of *L*-ascorbic acid in water at 180 °C. These CDs were classified as CQDs and had average particle sizes ranging between 1 and 4 nm. These CQDs were then annealed at 200–700 °C for 10 min [117]. Carbon chunks similar to those observed by Hou et al. [116] were observed at 250 °C. These were further developed into multi-layered sheets at T ≥ 300 °C. Further heating to 700 °C gave thin layered graphene-like sheets that lacked lattice fringes. The thin layered graphene-like sheets were found to have similar properties to traditional rGO and they were, thus, referred to as rGO (Figure 4a,b). In the study, the temperature was shown to be the main contributing factor that induced the transformation of CDs to rGO. Solid-state NMR studies revealed that an increase in temperature resulted in deoxygenation of the CDs, as shown by an increase in the C/O ratio. An increase in C species led to the generation of C-C bonds and layered sheets.

In contrast, Bharathi et al. reported that a metal catalyst was needed for the formation of graphene sheets from GQDs [118]. In their study, the GQDs were synthesized via “bottom-up” hydrothermal synthesis using citric acid and urea in water at 180 °C for 3 h. The obtained CD average particle sizes were between 2 and 5 nm. The graphene sheets were obtained by adding varying amounts of zinc ZnCl_2_ to the reaction mixture, while the reaction temperature was kept constant (Figure 4c,d). Upon the addition of 0.1 mol ZnCl_2_ to the mixture, the GQDs’ average size increased to ~10 nm. The addition of a larger amount of ZnCl_2_ (0.3 mol) gave ~100 nm particles, while micro-sized graphene-like sheets were formed when 0.5–0.7 mol ZnCl_2_ was added. The XPS analysis of the formed sheet materials did not show changes in the C/O ratio.

**Figure 5:**
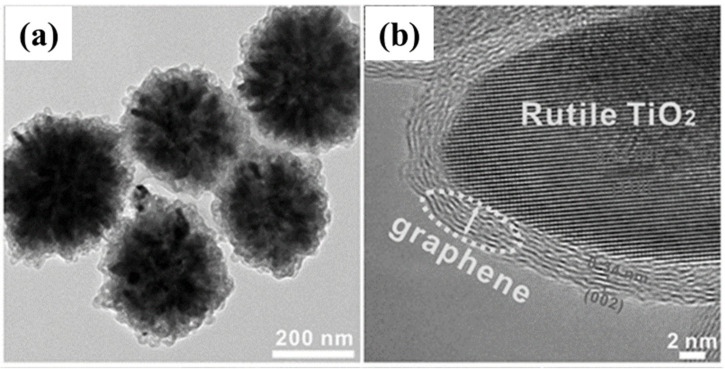
(**a**) TEM and (**b**) HR-TEM of graphene-rich petal-like rutile TiO_2_ from 0-D CDs and TiO_2_ precursors (adopted with permission from [119], 2016 WILEY-VCH Verlag GmbH and Co. KGaA, Weinheim).

In another study, Zhang et al. showed that CDs could be used as so-called “designer additives” in the synthesis of graphene-rich, petal-like rutile TiO_2_ [119]. In this study, the CDs were synthesized from acetone mixed with NaOH. The solution was left to react for 5 days at room temperature [17]. The CDs synthesized using this method were classified as CQDs and contained average particle sizes ranging between 1.5 and 3 nm. The CQDs were then added together with the TiO_2_ precursor (made from titanium(III) chloride) and CTAB (dissolved in n-pentanol and n-hexane) and reacted at 200 °C. The resulting product was further annealed at 800 °C for 2 h to give a graphene-rich, petal-like rutile TiO_2_ (Figure 5). It was reported that these CQDs formed petal-like structures that acted as the TiO_2_ template. The concentration of these CQDs affected the crystallite growth and resulting crystalline phases (rutile/anatase), as well as particle shapes of the TiO_2_. The TEM studies revealed irregular TiO_2_ products in the absence of CQDs. In the presence of the CQDs, the final annealed product indicated that the CQDs had been transformed into graphene layers that were wrapped around the TiO_2_ particles. Other similar studies have shown that CDs can also be assembled on various other porous materials, including zeolites, carbonaceous porous materials, porous graphitic carbon nitride, mesoporous SiO_2_ and metal–organic frameworks [80].

### 3.3. CDs Used as Precursors to Make 3-D Carbon Allotropes

Porous carbon frameworks (PCFs) are an interesting group of materials known for their high surface area and tunable surface chemistry. These materials have been applied in fields such as catalysis, separation, gas storage and sensing. The traditional synthesis of these materials includes “bottom-up” synthesis strategies using organic or bio-molecules and graphene nanoribbons, while the “top-down” methods include the use of metal–organic frameworks or carbides [120]. The use of CDs as carbon precursors provides another alternative synthesis strategy to make these materials.

**Figure 6:**
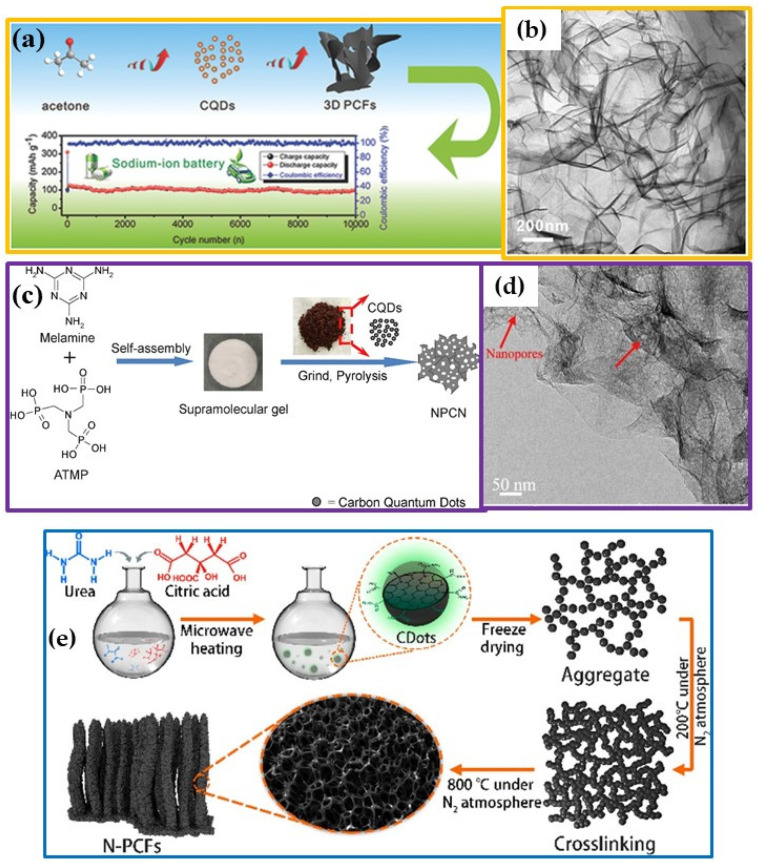
Schematic illustrations of the different synthesis methods used to convert 0-D CDs into 3-D (**a**,**b**) PCFs (reproduced with permission from [17], copyright 2015, Wiley-VCH), (**c**,**d**) N- and P-doped PCFs (reproduced with permission from [121], 2017 Elsevier Ltd.) and (**e**) N-PCFs (reproduced with permission from [122], copyright 2019, American Chemical Society).

Hu et al. synthesized PCFs (Figure 6a,b) using CDs as precursors. The CDs were synthesized using a simple “bottom-up” approach that involved mixing acetone and NaOH (5 days at room temperature). The obtained CDs had average particle sizes ranging between 1.5 and 3 nm, and were classified as CQDs [17]. The XPS results showed that these CDs contained ~83% C and ~17% O, with C-OH, C=C, C-H and C=O as functional groups. The PCFs were obtained by calcining these CDs at 400, 600 and 800 °C under argon for 2 h at each temperature. During the transformation of the CDs to PCFs, the authors noted an increase in the C% from ~83% (for CDs) to ~94% for the calcined samples. Their results also showed that the materials showed a sharp increase in specific surface area with the increase in calcination temperature. The mechanism of PCF formation from CDs was believed to involve a catalyst-assisted transformation.

Jiang et al. performed further modifications to Hu et al.’s method [17] to further alter the surface chemistry of the PCFs by doping them with nitrogen (N) and phosphorus (P) [121]. In this work, melamine and C_3_H_12_NO_9_P_3_ were used to form a supermolecular gel. The CQDs and supermolecular gel were then heat-treated at 800, 900 and 1000 °C under argon (Ar) for 1 h. The supermolecular gel acted as an N and P source and was also directly involved in the formation of the 3-D structure of the PCFs (Figure 6c,d). The authors also noted an increase in specific surface area with the increase in temperature, and noted that the doped PCFs were highly porous, with the pore volume increasing with the increase in treatment temperature, with a final pore volume of 0.542 cm^3^ g^−1^ obtained at 900 °C. Interestingly, the authors also reported that annealing the CQDs without a supermolecular gel at 800 °C yielded 2-D graphene-like nanosheets.

In another study, nitrogen-doped CDs were used as precursors for the synthesis of fibrous 3-D PCFs. The CDs were produced via the microwave-assisted irradiation of citric acid and urea in DMF at 500 W and 160 °C [122]. After purification, the resulting solution was freeze-dried to obtain CDs with approximate average sizes ranging between 3 and 5 nm. To achieve the 3-D PCFs, the CDs were subjected to heat treatment at 800 °C under N_2_ for 2 h (Figure 6e). The 3-D PCFs exhibited some graphitic characteristics, such as the lattice d-spacings of 0.12, 0.21, and 0.34 nm corresponding to the (002), (100) and (110) planes of a graphitic carbon, respectively. Additionally, the 3-D PCFs contained ~19% nitrogen content, as confirmed by the XPS analysis. Interestingly, the XPS results also showed that the CDs contained predominantly pyrrolic-N, while the 3-D PCFs contained mainly pyridinic-N and graphitic-N. The data suggest that the transformation of the 0-D CDs to 3-D PCFs is also accompanied by the conversion of the pyrrolic-N to pyridinic-N and graphitic-N.

**Figure 7:**
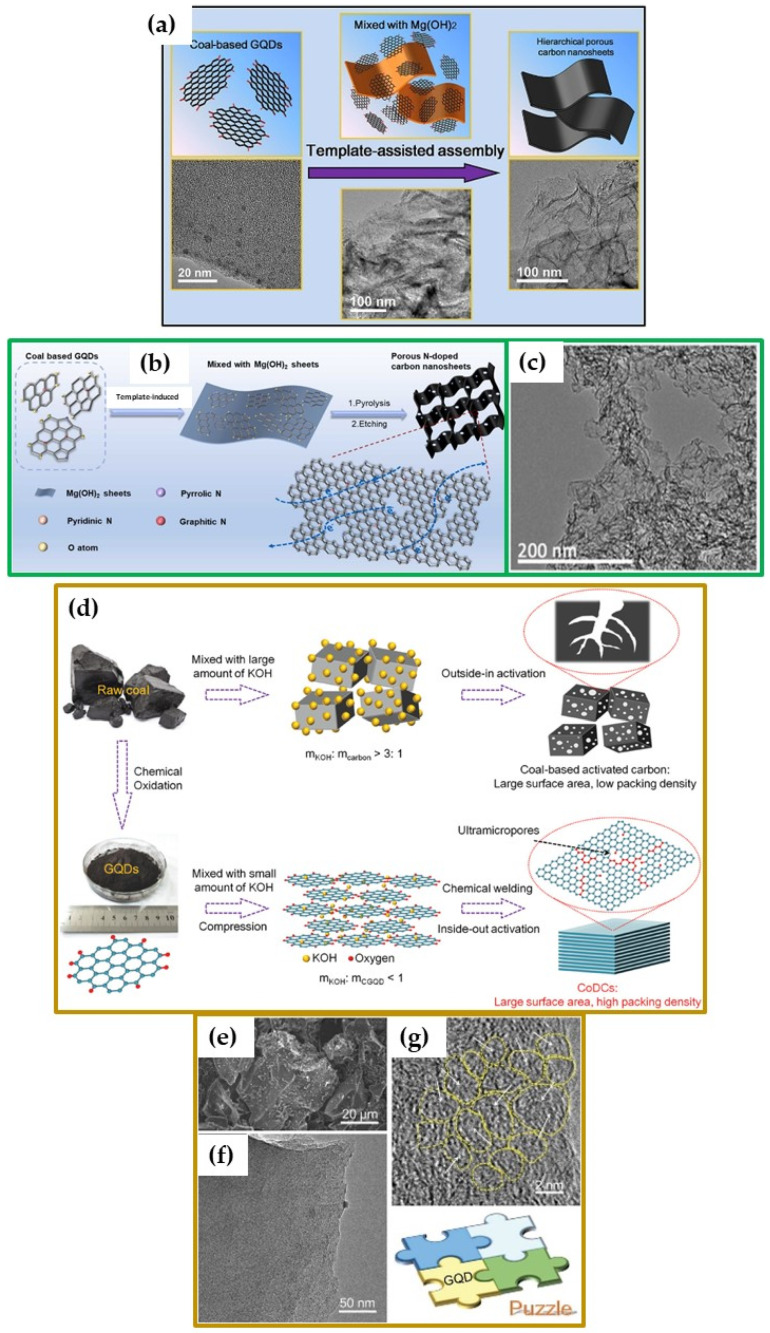
Coal-derived CDs used as precursors for the template-assisted synthesis of (**a**) PCFs (adopted with permission from [91], 2017 Elsevier Ltd.), (**b**,**c**) N-PCFs (reproduced with permission from [123], 2022 Elsevier B.V.) and (**d**–**g**) ultra-microporous carbon (adopted with permission [124], 2018 WILEY-VCH Verlag GmbH and Co. KGaA, Weinheim), as well as the TEM images of the resulting products.

Zhang et al. devised a different template-assisted method for the synthesis of PCFs from CDs. To make their CDs, they used a “top-down” acid treatment (with HNO_3_ and H_2_SO_4_) of bituminous coal [91]. The CDs were classified as GQDs and had average particle sizes in the 2–3 nm range. The transformation of GQDs to PCFs was done on a Mg(OH)_2_ template. The amount of Mg(OH)_2_ and the temperature (700–1000 °C) were varied to determine their effects on the resulting structure. The surface chemistry analysis revealed that these GQDs had abundant oxygen functional groups (~45%). As expected, increases in the annealing temperature and Mg(OH)_2_ amount decreased the oxygen content to ~7%. The authors showed that the synthesis of GQDs to PCFs was induced by the Mg(OH)_2_ template. Recently, Lei et al. were able to prepare nitrogen-doped GQDs (~2–3 nm) by decomposing bituminous coal fine powder in HNO_3_ and H_2_SO_4_. These GQDs were then mixed with the Mg(OH)_2_ template and heat-treated at 800–1000 °C for 1 h under an Ar atmosphere (Figure 7b,c) [123]. The optimum results were obtained for a sample prepared at 900 °C. The resulting 3-D porous framework contained carbon (87.7%), nitrogen (4.1%) and oxygen (8.2%). As with the study by Zhang et al., increasing the temperature decreased the oxygen content. When GQDs were heated at 900 °C without the Mg(OH)_2_ template, irregular block shapes were observed with a lower % carbon and nitrogen content but a higher oxygen content compared to the sample prepared with a Mg(OH)_2_ template.

In another study, GQDs (made from coal, diameter of ~2.96 nm) and potassium hydroxide at different ratios (0, 0.5 and 1), gave ultra-microporous carbons (Figure 7d–g) [124]. At a 0.5 ratio, the HRTEM images showed that the GQDs were joined together to form layers, but the individual GQDs could still be observed (Figure 7g). Similar observations were noted in the formation of supra-CDs (see Section 3.4). When a reactant ratio of 1 was used, a 3-D structured carbon with pore sizes of approximately ~20 nm was formed. When the GQDs were heated alone, 2-D layered materials were formed. As expected, the XPS data showed that the O/C ratio decreased from 0.51 for the GQDs to 0.11 when the 0.5 ratio was used.

Strauss et al. produced a porous 3-D turbostratic graphene (Figure 3g) by irradiating N-doped CDs with an infrared laser [125]. The N-doped CDs were synthesized via “bottom-up” microwave-assisted irradiation of citric acid and urea in water, which produced nanoparticles with average sizes in the range of 1–5 nm [126]. To form the porous 3-D turbostratic graphene, the CNDs were first annealed at 300 °C to give CND aggregates (~50 nm). These CND aggregates were then laser-irradiated to form a porous 3-D turbostratic graphene. The laser irradiation induced decarboxylation of the CNDs and catalyzed the C-C bond formation between the CND moieties. Gases such as CO_2_ were released during the laser irradiation, and this gave rise to the formation of the porous 3-D morphology of the graphene.

**Table 1:** A summary of the methodologies followed for the transformation of 0-D CDs into 1-, 2- and 3-D carbon allotropes.

No.	Carbon Dots			Assembly Process			
	Precursor(s)	Type	Average Size Range (nm)	Synthesis	Type of Carbon Allotrope	Dimensions (D)	Ref.
1	Graphene	GQDs	3–5	Assembled inside the anodic aluminum oxide via electrochemical deposition	GQD-NTs	1-D	[100]
2	Alanine and histidine	CQDs	~10	Pyrolyzed with ZIF-67 powder	N-CNTs	1-D	[101]
3	fullerene (C_60_)	CQDs	~7	Annealed under inert gas atmosphere	Layered sheets	2-D	[115]
4	acetaldehyde solution and NaOH	-	3–5	Mixed with NaH_2_PO_4_ and calcined under Ar gas atmosphere	carbon sheets	2-D	[116]
5	L-ascorbic acid	CQDs	1–4	Annealed under N_2_ inert gas atmosphere	carbon sheets (rGO)	2-D	[117]
6	citric acid and urea	GQDs	2–5	Hydrothermal synthesis in the presence of ZnCl	graphene sheets	2-D	[118]
7	acetone and NaOH	CQDs	1.5–3	Reaction with TiO_2_ precursors in an autoclave, then annealed under an Ar atmosphere	graphene	3-D	[119]
8	Acetone and NaOH	CQDs	1.5–3	Calcination under an Ar gas atmosphere	PCFs	3-D	[17]
9	Acetone and NaOH	CQDs	-	Pyrolyzed with melamine and C_3_H_12_NO_9_P_3_ under Ar atmosphere	N-, P-PCFs	3-D	[121]
10	Citric acid and urea	-	3–5	Freeze-dried and calcined under N_2_ atmosphere	Fibrous N-doped PCFs	3-D	[122]
11	Bituminous coal	GQDs	2–3	carbonized on a Mg(OH)_2_ template	Hierarchical PCFs	3-D	[91]
12	Bituminous coal	GQDs	2–3	carbonized on a Mg(OH)_2_ template	N-doped PCFs	3-D	[123]
13	Bituminous coal	GQDs	~3	Carbonized with potassium hydroxide	Ultra-microporous carbons	2-/3-D	-
14	Citric acid and urea	CNDs	1–5	Thermolyzed in a tube furnace and laser irradiation with 40 W CO_2_ laser	turbostratic graphene	3-D	[125]
15	Citric acid and aqueous ammonia	N-, S-CDs	~4	Pyrolyzed on Fe_2_O_3_, NaCl and SiO_2_ spheres templates	microrods, carbon sheets and PCFs	1-/2-/3-D	[127]

GQDs: graphene quantum dots; GQD-NTs: graphene quantum dot nanotubes; CQDs: carbon quantum dots; N-CNTs: nitrogen nanotubes; 2-D: two dimensional; rGO: reduced graphene oxide; 3-D: three dimensional; PCFs: porous carbon frameworks; N-, P-PCFs: nitrogen- and phosphorus-doped porous carbon frameworks; CNDs: carbon nanodots.

**Figure 8:**
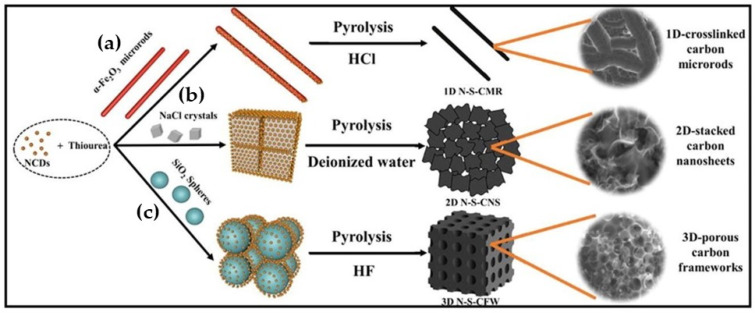
A schematic illustration of the template-assisted conversion of CDs into N- and S-doped (**a**) 1-D microrods, (**b**) 2-D carbon sheets and (**c**) 3-D PCFs (reproduced with permission from [127], 2021 Elsevier B.V).

Another interesting study was reported by Cheng et al., who were able to transform 0-D nitrogen-doped CDs into 1-, 2- and 3-D carbon structures using different metal templates [127]. In their study, citric acid and aqueous ammonia were used to synthesize nitrogen-doped CDs with an average particle size of 4 nm. These nitrogen-doped CDs were then mixed with thiourea and a template (Fe_2_O_3_ rods, NaCl and 3-D stacked SiO_2_ spheres) to form nitrogen and sulfur co-doped structures (Figure 8). The mixtures were first freeze-dried, then heated at 300 °C and finally pyrolyzed at 900 °C for 4 h under a N_2_ atmosphere. After the removal of the templates, it was observed that nitrogen and sulfur co-doped 1-D microrods (width ~100 nm), 2-D stacked carbon nanosheets and 3-D porous carbon frameworks (diameter of the 3-D stacked spheres = ~200 nm) had been made. Interestingly, the nitrogen and sulfur contents (XPS atomic abundance: N = 5.58% and S = 1.64%) were higher in the 2-D carbon structure when compared to the other structures, with pyrolic-N being the dominant nitrogen configuration present. It was observed that the pyrolysis temperature played a significant role in the transformation of these nitrogen-doped CDs on differently structured templates, similar to the reports mentioned previously.

### 3.4. Other CD Assembly Products

The other assembled structures produced from the CDs included the supra-CDs. Supra-CDs are typically non-permanent nanostructures of CDs formed by inter-particle interactions between CD nanoparticles via strong or weak bonding forces between surface functional groups. The formation of supra-CDs has been used for the broadening of the PL absorption spectra of the CDs [128,129,130]. A variety of CDs have been used to produce these supra-CDs.

**Scheme 1:**
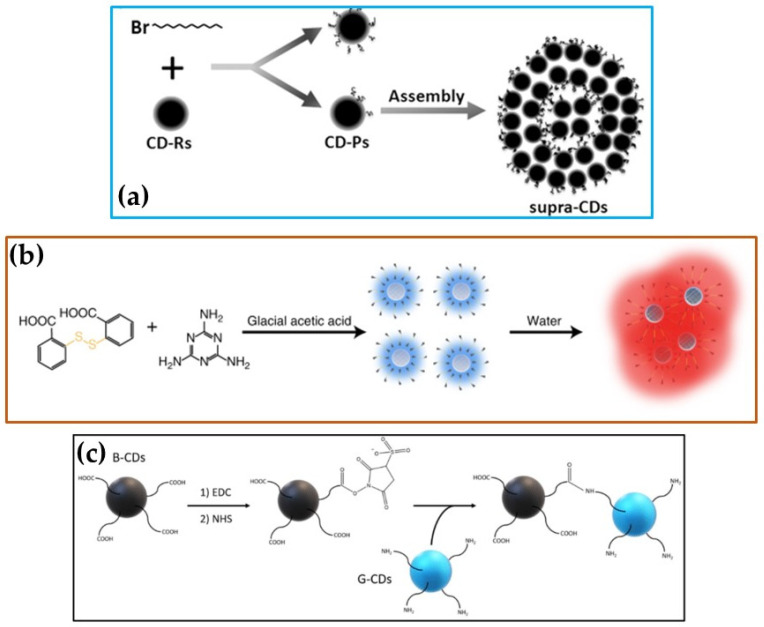
Schematic depiction of the conversion of CDs to supra-CDs (**a**) in toluene (adopted with permission [128] from WILEY-VCH Verlag GmbH and Co. KGaA, Weinheim, 2015) and (**b**) in dimethylformamide (reproduced with permission from [130], Springer Nature, 2019), and (**c**) the covalent conjugation reaction of CDs (adopted with permission from [132], 2020 Elsevier BV).

Lou et al. prepared CDs from a “bottom-up” synthesis method using citric acid and urea as the starting precursors. The modification of the CDs with 1-bromododecane gave partially functionalized CDs with average particle sizes ranging between 1 and 5 nm, with an amphipathic surface. In toluene, these partially functionalized CDs self-assembled through amphipathic interactions to give nanoclusters with sizes of 150–400 nm (see Scheme 1a), described as supra-CDs [128]. Li et al. showed that CDs made from citric acid and urea could self-assemble without attaching alkyl chains onto the CDs’ surfaces. In their study, they also obtained CNDs with sizes ranging between 1 and 5 nm. When the CNDs were left under 60% humidity for 7 days, supra-CDs were formed. These supra-CDs were smaller when compared to the ones obtained by Lou et al., with sizes ranging between 5 and 25 nm [129].

Another study by Yang et al. showed that supra-CDs could be formed via the self-assembly of CDs obtained from melamine and dithiosalicylic acid in acetic acid. The CDs had average particle sizes ranging between 4 and 10 nm. When placed in dimethylformamide, these CDs interlinked with each other to form supra-CDs (see Scheme 1b) with sizes around 56 nm [130]. Hou et al. also showed that the CDs prepared via the “top-down” method could be used to make supra-CDs. In this study, CDs were synthesized by breaking down ultrafine coal pitch with formic acid and hydrogen peroxide. The obtained CDs were then dispersed in the surface microchannels of balsa wood. After drying at 60 °C, it was observed that the pore sizes of this wood decreased from 14.7 nm to about 1 nm, signifying that the CDs had self-assembled to form supra-CDs inside the pores of the wood [131]. It can also be assumed that the interconnected CQDs that were formed at the ice–crystal–water interface in a study by Chen et al. [115] (described above) were also another example of supra-CDs.

The conjugation of differently functionalized CDs is another way of building dimensional carbon allotropes. In these reactions, chemical linkers are used to react with specific chemical groups on the CD surface to be conjugated, and these reactions form covalently coupled nanostructures [132,133]. For example, Zhou et al. performed an amidation reaction between “black CDs” (B-CDs) and “gel CDs” (G-CDs) [132]. The B-CDs were obtained via the “top-down” method involving breaking down carbon powder into H_2_SO_4_ and HNO_3_, while the G-CDs were made in a “bottom-up” reaction, which involved reacting ethylenediaminetetraacetic acid with citric acid. The B-CDs were predominantly functionalized with -COOH groups, which were activated and stabilized using N-ethyl-N’-(3-(dimethylamino)propyl)carbodiimide/N-hydroxysuccinimide to form intermediates that were subsequently reacted with –NH_2_ groups found on the surfaces of the G-CDs to form a conjugate (see Scheme 1c below). The reactions were performed by first dispersing the CDs in phosphate-buffered saline solution (pH 7.4), then adding them together and allowing them to react overnight while stirring. Different B-CD-to-G-CD ratios were used in the reaction to produce different nanostructures. For example, when the B-CD-to-G-CD ratio was 5:3, the particles appeared to merge to form a figure-eight shape. When the ratios were changed to 5:100 and 5:300, nanofibers and some nanosheets were formed [132]. The conjugation reactions produced similar results to the heat-treated systems. However, in these reactions, higher-dimensional carbon structures were achieved by changing the ratio of the starting materials. In some instances, the CDs self-conjugated to form larger CD particles. For example, CDs (~2 nm) with predominantly –COOH and some –NH_2_ were able to self-conjugate to form larger particle sizes (68 ± 30 nm). Conjugation reactions were also used to link the CDs with other species such as metal-based quantum dots [134] and drug molecules [135].

## 4. Mechanism of Formation of the Dimensional Carbon Allotropes from CDs

From the discussion above, it is noted that the CD assembly process requires the alteration of surface functional groups, which leads to the transformation of the physical structure of the CDs.

### 4.1. Chemical Transformation

As mentioned in the introduction, CDs are terminated by functional groups, e.g., O- and N-based functional groups, depending on the type of synthesis procedure followed to make them. The CD assembly process appears to be highly dependent on the reduction or removal of surface functional groups, especially in a deoxygenation process. This reduction or removal leads to an increase in the C species, which then react to form C-C bonds and the larger carbon allotropes [17,117]. The XPS analysis results are usually used to confirm the functional group’s removal or transformation. The evidence of this transformation is mostly shown by an increase in the C atomic %, accompanied by a decrease in the O atomic %. Additionally, there is also evidence that some of the O-based surface functional groups are transformed from one bonding configuration to another. For example, Strauss et al. noted that during the transformation of CNDs to porous 3-D turbostratic graphene, some of the C-O-H groups from the CNDs were transformed into C-O-C groups when the porous 3-D turbostratic graphene was formed [125].

In the case of the assembly of N-doped CDs to dimensional allotropes, some authors have shown that there are similar changes in the N-functional groups as with the O-functional groups. In most of the reported syntheses, the N-doped CDs are synthesized by using urea as a nitrogen source. For example, Strauss et al. synthesized N-doped CDs using citric acid and urea and the authors reported that the N-H functional groups from the CDs were removed when the CDs were laser-irradiated [125]. In another example where N-doped CDs were also synthesized from citric acid and urea, Zhang et al. noted that the transformation of the N-doped CDs to PCFs (at 800 °C) led to a decrease in the N atomic %, as well as the conversion of N-pyrrolic functional groups in the CDs to graphitic- and pyridinic-N in the PCFs [122]. The transformation of N-pyrrolic functional groups in the CDs to graphitic- and pyridinic-N after the heat treatment was also observed in the transformation of N-doped CDs from *L*-ascorbic acid and urea to 2-D graphene-like sheets at temperatures ranging between 200 and 700 °C [136]. Interestingly, when the N-doped CDs from citric acid and urea were reacted in the presence of a Zn catalyst (different concentrations of the catalyst at 180 °C), the resulting 2-D sheets contained only graphitic-N [118].

Hou et al. synthesized N- and P-incorporated PCFs from CDs and noted a decrease in the O-based functional groups after heat treatment. The authors also noted an increase in the P atomic % with the increase in heat treatment from 400 to 900 °C [116]. However, it was not clear whether or not the P-configuration changed with the temperature or not. Investigations into how other heteroatoms are affected by the heat treatment or catalyst-induced transformation of the CDs are needed.

The functional groups are typically removed or transformed using a catalyst or thermal procedures by knitting together the sp^2^/sp^3^ carbon layers of the CDs to form the 1-, 2- and 3-D carbon allotropes. Thus, the study by Hou et al. showed that both the temperature and metal catalyst were needed for the transformation of CDs to larger carbon allotropes [17,116]. However, other studies showed that only thermal procedures were needed to convert CDs and N-doped CDs to graphene nanosheets [117,136], while Bharathi et al. formed layered sheets by varying only the catalyst concentration [118]. In contrast, the synthesis of supra-CD studies suggested that the CDs can assemble spontaneously [129] merely by adding them to a solvent [128,130]; however, these are not permanent structures.

In the process of CD conjugation, the surface functional groups on the CDs undergo similar reactions to those of small organic molecules and form covalent bonds, which join together with CD particles to form different carbon structures [132,133].

### 4.2. Physical Transformation

The resulting carbon morphology appears to be dependent on the template used to make the carbon material from the CDs. If a template is used, the CDs will assemble into the shape of the template. For example, the transformation of CDs to CNTs requires the use of a tubular-shaped template to achieve the hollow tube morphology [100,101]. It is not yet clear what types of morphologies will result from the transformation of the CDs synthesized in the absence of a template. However, Jiang et al. showed that the CDs that were heat-treated at 800 °C in the presence of a supermolecular gel template yielded 3-D PCFs, while without this supermolecular gel template 2-D graphene-like nanosheets were obtained [121].

Also of note is the observation that the material that forms the CDs can influence the final structure. Thus, when CDs were produced using acetone as one of the precursors, excess acetone molecules were entrapped into the layered sheets. Their presence or removal during high-temperature annealing precluded the nanolayers from stacking on top of each other, as found in graphene and graphite [17,121]. It will be interesting to establish whether the absence of surface heteroatoms (as in hydrophobic CDs) will still lead to the CDs’ structural transformation in a solvent (as in supra-CDs), under high temperature and with a catalyst.

It is still not clear whether the CD identity (i.e., GQDs, CQDs, CNDs and CPDs) affects the structure of the final carbon allotrope that is formed. This arises since the classification of CDs is based on their synthesis, not on the products they form [14]. For example, only CDs obtained from the exfoliation of graphitic materials are called GQDs [137]. However, GQDs can also be obtained from non-graphitic natural starting materials such as durian [138], with crystallinity levels as high as those obtained from graphitic materials. Over the years, the better characterization and interpretation of the CDs properties has led to better-defined categories of CDs (GQDs, CQDs, CNDs and CPDs), with the classification not only being based on their synthesis but also their physicochemical properties as well [5,14]. Even so, the current classification approach is still used inconsistently [5,14]. Therefore, it is possible that some of the CDs used in earlier studies for the formation of larger carbon allotropes from CDs were misclassified. Further studies devoted to understanding the relationship between the identity of CDs and the type of carbon allotrope formed after their thermal- or catalyst-mediated transformation are needed. Our suggestion for this would be to take advantage of machine-learning-based techniques.

Recently, machine-learning-based techniques have been used to further our knowledge base and understanding of CDs. More importantly, machine learning has been used to gain a further understanding and build experimental models using data and algorithms to correlate the structure–property relationship of CDs [139]. Thus, machine-learning-based techniques have been used to develop strategies that allow the synthesis of CDs with targeted optical properties [140,141], optimized quantum yields [142] and high selectivity in gas sensing [143]. Since machine learning is already shedding some light on the structure–property relationship, it is possible that this tool can potentially predict the identity of the CDs (i.e., GQDs, CQDs, CNDs and CPDs) and the type of dimensional carbon formed after heat treatment or catalysis reactions, and potentially can also help control the amount of dopant (e.g., N-, P-doping) in the final carbon structure.

## 5. Application of the Assembled CDs Products

A variety of literature reports have shown that carbon nanomaterials including CDs can be applied in clean and sustainable energy production. Similarly, studies have shown that the new 1-, 2- and 3-D carbon allotropes *derived from CDs* also show great potential for application in energy conversion and storage [14,20]. Thus, these materials have been applied in electrode materials, separators and electrolytes for the fabrication of a new generation of batteries [14,20]. For example, the N-CNTs derived from N-CDs proved to be useful in the study of metal-free electrocatalysts for oxygen reduction reactions (ORRs). Thus, N-CNTs derived from N-CDs showed high electrocatalytic performance, with a high positive potential of 0.80 V and peak current density of 1.21 mA/cm^−2^. This high performance was attributed to the highly porous structure, high surface area and enhanced structural features obtained due to the use of N-CDs. This performance was shown to be better than the commercial Pt/C catalyst used under the same conditions [101]. Similarly, the 3-D P- and N-functionalized PCFs derived from CDs showed a high positive potential of 0.93 V and peak current density of 4.49 mA/cm^−2^ as compared to a commercial Pt/C [121].

The 2-D carbon allotropes derived from CDs have previously been used in supercapacitors. Chen et al. produced carbon layers that showed a high electrode density (1.23 g cm^−3^), high volumetric capacitance (157.4 F cm^−3^) and good rate capability (0.66 F cm^−2^) behavior in 6 M KOH aqueous electrolytes. This high performance was also attributed to the highly porous nature of the carbon allotropes used in the fabrication of the supercapacitor electrodes [115].

The 3-D PCFs synthesized from CDs have a high surface area, appropriate pore sizes and allow the easy incorporation of different dopants to provide good storage sites for sodium or lithium ions in sodium or lithium ion batteries, with increased conductivity when used as high-density supercapacitor electrodes. The full scope of these results may be found in other reviews [20,144]. Additionally, due to the properties mentioned, these materials have also been successfully used as catalysts for oxygen reduction reactions [123,127].

The supra-CDs have not been extensively explored. However, they are known to cause significant modifications of the luminescence properties of the CDs and to subsequently expand the scope of the potential of the CDs in different applications, particularly in the medical fields. These materials have shown potential for use as fluorescence nanomaterials for biological applications [128,129]. The supra-CDs can be used in near-infrared photothermal applications [129,131]. Other future potential uses of these supra-CDs include their application in optical communications [128].

Conjugated CD structures have shown promising applications in nanomedicine and as drug nanocarriers. Conjugated CDs have been created to be able to deliver drugs through the blood–brain barrier to the central nervous system. For example, the B-CD and G-CD conjugates discussed earlier have properties that make them favorable as drug delivery nanostructures. These properties include their good aqueous stability and bone-targeting ability (B-CDs) and their rich –COOH and -NH_2_ surface functional groups, which can assist during diffusion into the blood–brain barrier (G-CDs) [132]. These conjugated CDs can, thus, be used as drug carriers for central nervous system-related diseases [132,133].

## 6. Conclusions and Recommendations

The conversion of one carbon allotrope into another leads to materials with novel physicochemical properties that can be used for numerous applications. The transformation of CDs to 1-, 2-, and 3-D carbon allotropes as discussed here is no exception. Many reports have shown that these novel CD conversion processes represent facile synthesis strategies to make new and known dimensional carbon allotropes. Additionally, since CDs can be derived from a variety of cost-effective and recyclable carbons using simple synthesis strategies, these novel syntheses could potentially be a more cost-effective route for the synthesis of these larger carbon allotropes.

The assembly of CDs takes place via the interactions of the functional groups on the CD surface. The oxygen functional groups specifically are the ones driving this process. For the formation of supra-CDs, these interactions can be easily destroyed by redispersion, while in the 1-, 2- and 3-D carbon allotrope the functional groups are chemically removed or reduced, leading to a permanent merging of the CD nanoparticles into a larger carbon allotrope. The latter is achieved by using heat treatment or metal catalysts (e.g., Na^+^, Zn^2+^). It is important to further investigate the effect of each parameter separately and in detail. The studies on carbon nanomaterials have shown that they decompose at lower temperatures in the presence of metal catalysts. Therefore, it is possible that the 1-, 2- and 3-D carbon allotropes can be obtained at milder temperatures.

A current limitation to the formation of the larger structures using CDs relates to the size and surface chemistry associated with the CDs. The nanosized CDs are readily dispersible in solvents, and this makes their purification and collection using filtration techniques difficult. Further, the synthesized CDs have a range of sizes and ‘molecular weights’, and these factors will impact on their surface chemistry and use.

Thus far, not enough data have been generated to establish the CD identities (GQDs, CQDs, CNDs and CPDs) and their effect on the formation of the assembled carbon allotropes. Further studies on this topic still need to be conducted. Machine-learning-based techniques can potentially assist in this regard. The results, however, show that the carbon morphology is affected by using a template or by the nature of the starting materials. For example, CDs derived from acetone form 3-D carbon materials under thermal treatment because they contain excess acetone, which is removed under heat treatment, and this gasification alters the shape of the formed sheets from 2-D to 3-D. Without both gas and heat treatments, the resulting morphology is most likely to be a 2-D graphene-based sheet structure.

It is worth noting that there is an emergence in the literature of metal-free carbon composites involving CDs, such as CD–graphene and CD–CNT composites. These composites have shown that there is great synergy between the two carbon allotropes, which results in materials with improved properties for applications in energy materials and biological studies. The current studies have shown that these composites are, however, made by synthesizing the carbon allotropes separately and later mixing them to form a final composite product. This review shows that it is possible to adopt a one-step synthesis approach to make these composites. For example, using methods such as the “bottom-up” carbonization of citric acid, researchers were able to produce 2-D allotropes that still contained some residual CDs within the layers, making their final products carbon composites. There is potential for other synthesis methods to be tuned in such a way that other carbon composites can be formed as well. It will also be worthwhile to conduct studies to compare the CD-derived allotropes and allotropes derived from conversional synthesis methods in terms of their physicochemical properties and performance in different applications.

These new carbons formed from CDs have already been exploited for use in capacitors and in nanomedicine. Further uses can be envisaged in these areas, as well as for uses in solar cell devices and as sensors due to their size and peculiar physical properties (e.g., fluorescence). It appears that the small differences in the new carbons—determined by their source—have an impact on their properties. Thus, future studies could establish (i) the role of the type of CD (see Figure 1) on the final carbon product, (ii) the role of the functional groups in generating the new larger carbons, (iii) the advantages and disadvantages of these carbons made from CDs compared with equivalent carbons made using classical ‘top-down’ methods and (iv) whether they can be used in less traditional arenas such as in dielectrics and heat dissipation systems.

## Data Availability

Not applicable.

## Abbreviations

Abbreviation	Meaning
CDs	Carbon dots
CNTs	Carbon nanotubes
GNSs	Graphene-based nanosheets
PCFs	Porous carbon frameworks
CNOs	Carbon nano-onions
CSs	Carbon spheres
HCSs	Hollow carbon spheres
GQDs	Graphene quantum dots
CQDs	Carbon quantum dots
CNDs	Carbon nanodots
CPDs	Carbonized polymer dots
GO	Graphene oxide
GNRs	Graphene nanoribbons
